# Dietary adequacy and nutritional status of Meitei community of Manipur, Northeast India

**DOI:** 10.1111/mcn.13046

**Published:** 2020-12-21

**Authors:** Bidyalakshmi Loukrakpam, Ananthan Rajendran, Radhika S. Madhari, Naveen Kumar Boiroju, Thingnganing Longvah

**Affiliations:** ^1^ Food Chemistry Division ICMR‐National Institute of Nutrition Secunderabad India; ^2^ Division of Maternal and Child Health ICMR‐National Institute of Nutrition Secunderabad India; ^3^ Division of Biostatistics ICMR‐National Institute of Nutrition Secunderabad India

**Keywords:** anthropometry, food consumption, mean probability of adequacy, micronutrients, nutritional status, stunting, underweight

## Abstract

Meitei is the main ethnic community that belongs to the north‐eastern state of Manipur in India. This community is bestowed with rich biodiverse resources with indigenous foods still form an integral part of their diet. However, limited data on the food and nutrient consumption as well as nutritional status of this community are available. This study was carried out on the children, adolescents and women of reproductive age (WRA) of this community from 12 villages, to determine their food consumption pattern and nutritional status. Basic anthropometry and 24‐h dietary intake assessment was conducted. The prevalence of underweight was 27%, stunting was 45% and wasting was 12% in children below 5 years. Stunting was observed among 34% of children 5–17 years of age. About 7% of WRA were undernourished, while 28% were overweight or obese. About 55% of 1–7 year‐old children had mean probability adequacy of 12 micronutrients <0.5, and the adequacy of individual micronutrients namely vitamin A, E and calcium were low. Dietary determinants such as low dietary diversity score, dietary species richness and the lowest tertiles of different food groups' intake (except for sugars, fish and sea foods and spices and condiments) predicted micronutrient inadequacy. In addition to a high prevalence of undernutrition in children and adolescents and overnutrition in WRA, the effect of various dietary determinants on micronutrient adequacy in the study group of the Meitei community are reported.

**Key messages**

- Undernutrition, stunting and wasting were prevalent in the children below 5 years, while overweight and obesity was more prevalent than chronic energy deficiency in women of reproductive age (WRA).
- Dietary determinants such as low dietary diversity score, dietary species richness and the lowest tertiles of different food groups' intake predicted micronutrient inadequacy.
- Most of the individuals had an imbalanced dietary intake with higher portion of energy coming from carbohydrate and very low from fat.
- The risk of inadequacy was very high for vitamin A, E and calcium among children and adolescents and that of vitamin A and E among WRA.

## 1 INTRODUCTION

The north‐eastern region of India, which comprises the seven states, forms an integral part of the Indo–Burma biodiversity hotspot of global significance (Myers, Mittermeier, Mittermeier, Da Fonseca, & Kent, 2000). This region is topographically isolated and has different socio‐cultural practices from the rest of the country (Singh, Alagarajan, & Ladusingh, 2015). Manipur is one of the seven states and comprises an area of 22,327 km^2^, out of which, 17,219 km^2^ is forest‐covered area (Forest Survey of India, 2015). Geographically, this state can be divided into two parts, namely, lowland valleys and hilly areas. The agriculture sector has a vital place in the economy of Manipur, and 53% of the workers in Manipur are engaged as cultivators and agricultural labourers (Government of Manipur, 2017).

Meitei, an Indo–Mongoloid group speaking the Tibeto–Burman language, is one of the indigenous communities of the sub‐Himalayan state of Manipur. The community is confined mainly to the lowland valley located in the central part of the state, and they have a long history of preserving their rich culture and traditions (Singh & Huidrom, 2013). From the time immemorial, the Meiteis share an inseparable relationship with its diverse bio‐resource system for livelihood, religious rituals, food, medicine and so forth (Singh, 2011; Singh & Singh, 1996). In spite of being a patriarchal society, Meitei women take a crucial role in their families' economy by participating in trade and commerce. They also take an active part in various affairs of the state besides performing household works (Sircar Manjushri, 1984).

According to the National Family Health Survey (NFHS 4) 2015–2016, among the below 5‐year‐old children in Manipur, 30% were stunted, 7% wasted, 2% severely wasted and 14% underweight. Even though these statistics are better than the national data, there has been an increase of overweight and obesity in adult women (26%). At the same time, 26% of the adult women are affected by anaemia. Without question, nutrition plays a major role in maternal and child health, and it is widely recognized that optimum nutrition in early life is the foundation for long‐term health. Assessment of food intake and nutritional status of a community is an important step in order to inform strategies to improve the nutritional status of a community (Gueri & Pena, 1998). A few studies have been reported on the nutritional status of Meitei children (Gaur & Singh, 1994; Singh & Devi, 2013), and very limited data are available on the food and nutrient intake of Meitei community.

Understanding the biodiverse food system of indigenous people is important because it contributes to the food system sustainability and global food biodiversity protection (Burlingame & Dernini, 2010; Kuhnlein, 2015). Considering the importance of indigenous food system, a study was carried out on the food system and its nutritional implications of the Meitei community focusing on the food biodiversity and development of food composition database of indigenous foods used in this community (Loukrakpam, Rajendran, Chyne, & Longvah, 2019). The data reported here are part of a study, which emphasized on the food availability, accessibility, consumption pattern and nutritional status of the indigenous Meitei community. The information presented here will help to understand the adequacy of food and nutrient intake and prevalence of malnutrition among children, adolescents and WRA of the Meitei community.

## 2 METHODS

### 2.1 Study design and sampling

From the lowland of Manipur, two districts namely Thoubal and Bishnupur were randomly selected for this study. The villages of these two districts were arranged based on the population from the least to highest and divided into quartiles. Equal number of villages was selected from each quartile by systematic random sampling, and a total of 12 villages were selected from the two districts for this study (Table S1). The sample size (690) was calculated using stunting prevalence (30%) among children under 5 years in Manipur (NFHS‐4) with a 95% confidence level and 5% precision and design effect of 2. A list of households in all 12 selected villages was obtained from the respective village head. From the list, every fourth consequent household was selected by systematic random sampling, and a total of 1,920 households were covered for anthropometry measurement. By covering 1,920 households, the estimated total number of children under 5 years to be covered would be 864 assuming a family size of 4.5 (National Nutrition Monitoring Bureau [NNMB], 2012) and considering 10% of the population to be children under 5 years (Census, 2011), which would cover the calculated sample size. Household dietary intake survey was carried out every eighth consequent household among the households covered in the anthropometry measurement, which derived up to 240 households.

### 2.2 Anthropometric measurement

Basic anthropometric measurements, that is, length/height and weight were measured up to the nearest 1 mm and 100 g using *seca* (417) infantometer/*seca* (213) stadiometer and weighing scale *seca* (803), respectively (Jelliffee & Jelliffee, 1990). The individuals covered for anthropometry measurements were categorized into below 5 years (*n* = 701), 5–7 years (*n* = 604), 8–12 years (*n* = 852), 13–17 years (*n* = 497) and women of reproductive age (WRA) (18–49 years) who were also nonpregnant and nonlactating (*n* = 1961).

Standard deviation (SD) classification recommended by World Health Organization (WHO, 2006) was followed to categorize the children below 5 years into different grades of nutritional status. The Z‐score of weight‐for‐age, height‐for‐age and weight‐for‐height below −2SD from the median of the reference population were defined as underweight, stunting and wasting, respectively, and below −3SD was considered severe (WHO, 2006). Children of the age between 5 and 17 years were categorized into nutritional grades using body mass index (BMI) for age Z‐scores (BAZ) and height for age Z‐scores (HAZ) following WHO reference values (Onis et al., 2007). Similarly, BAZ and HAZ below −2 Z‐score were defined as thinness and stunting, respectively. However, BAZ greater than 1 Z‐score, and 2 Z‐score was considered overweight and obesity, respectively. BMI was calculated for the WRA, and classification recommended by WHO was followed (WHO, 2000). BMI less than 18.5 kg/m^2^; between 25 and 29.9 kg/m^2^; and greater than and equal to 30 kg/m^2^ was used to define chronic energy deficiency, overweight and obesity, respectively.

### 2.3 Dietary intake assessment

A single day 24‐h recall was carried out using a validated questionnaire, 12 different sizes (volumes) of standardized cups, two spoons and kitchen weighing scale (*seca* 852) with precision of 1 g to acquire detailed information on the food intake of all the individuals in households. Details on quantities of raw ingredients used for preparation, cooking methods, the total amount of food cooked and its subsequent consumption by the individuals in the household were obtained from the caregiver responsible for food preparation and distribution in the household. Later, the raw food quantities consumed by the individuals is calculated from the cooked food consumed using the following equation.

Quantity of raw food ingredient consumed = (Quantity of raw food ingredient cooked/Total quantity of cooked food) * Quantity of cooked food consumed.

Then the nutrient composition of the foods consumed by the individuals was computed using Indian Food Composition Tables (Longvah, Ananthan, Bhaskarachary, & Venkaiah, 2017); nutrient values of Indian foods (Gopalan et al., 1989) and food composition of indigenous foods (Loukrakpam et al., 2019). For the dietary intake, the individuals covered were categorized into 1–7 years (*n* = 130), 8–12 years (*n* = 82), 13–17 years (*n* = 98) and WRA (*n* = 259). The pregnant and lactating women were not included as the sample size was very small.

### 2.4 Estimation of adequacy of nutrients

The percentages of energy consumption from protein, fat and carbohydrate were calculated and compared with acceptable macronutrient distribution range (AMDR) (WHO, 2003). Estimated energy requirements (EERs) were calculated for children (Food and Agriculture Organization/WHO/United Nations University, 2004) and WRA (Indian Council of Medical Research, 2010).

The probability of adequacy (PA) of the 12 micronutrients was calculated using estimated average requirements (EARs) recommended by Institute of Medicine (IOM), National Academies, Food and Nutrition Board, USA (Del Valle, Yaktine, Taylor, & Ross, 2011; Food and Nutrition Board IOM, 2005; IOM, 1997; IOM, 1998; Monsen, 2000; National Research Council, 2002). The SD of the requirements was obtained using coefficient of variation (CV) of the requirements (WHO, 2005) applying SD = CV * EAR. The EAR for iron was adjusted with 10% bioavailability while EAR for zinc (refined/mixed) was used as recommended by the International Zinc Consultative Group (IZiNCG, 2004). For the rest of the micronutrients, the PA was calculated using EAR directly as given by IOM. The PA for 12 micronutrients was calculated using CDFNORM function in SPSS. The PA determines the probability that the individual's nutrient intake meets the requirement (EAR). Mean PA (MPA) was determined as the mean of the PA of the 12 micronutrients, where and MPA <0.5 was used to determine the prevalence of micronutrient inadequacy (Becquey & Martin‐Prevel, 2010).

### 2.5 Dietary diversity score and dietary species richness

The dietary diversity score (DDS) and dietary species richness (DSR) were calculated from the 24‐h diet recall. DDS was calculated as the number of food groups consumed through the diet survey. The DDS was based on 13 food groups as listed in Table S3 (Radhika, Swetha, Kumar, Krishna, & Laxmaiah, 2018), and a minimum of 15 g of food consumed was considered as cut off for the inclusion in DDS (except for children under 2 years). DSR was calculated with reference to Lachat et al. (2018), which is the count of different species (plants and animals) consumed by each individual in a day.

### 2.6 Statistical analyses

Statistical analysis was carried out after duly satisfying all data scrutiny procedures with SPSS® for Windows® version 19.0. The food and nutrient intake data were skewed, so the daily intake of food groups and nutrients among the different age groups are presented as median with interquartile range (IQR), that is, 25th (P_25_) and 75th (P_75_) percentiles. Comparison of the median values across the different age groups was carried out by Kruskal–Wallis test while for the mean values, one way ANOVA was used with post hoc least significant difference test for multiple comparisons. Chi‐squared (*χ* ^*2*^) test was used for testing the association between categorical variables. Logistic regression analysis with crude and adjusted odd ratios (AOR) (95% confidence interval [CI]) was performed to identify different determinants of micronutrient inadequacy (MPA <0.5). The level of significance was considered at *p* < 0.05.

### 2.7 Ethical considerations

Ethical clearance was obtained from Institutional Ethical Committee of National Institute of Nutrition, Hyderabad (14/II/2017).

## 3 RESULTS

### 3.1 Anthropometry

**FIGURE 1:**
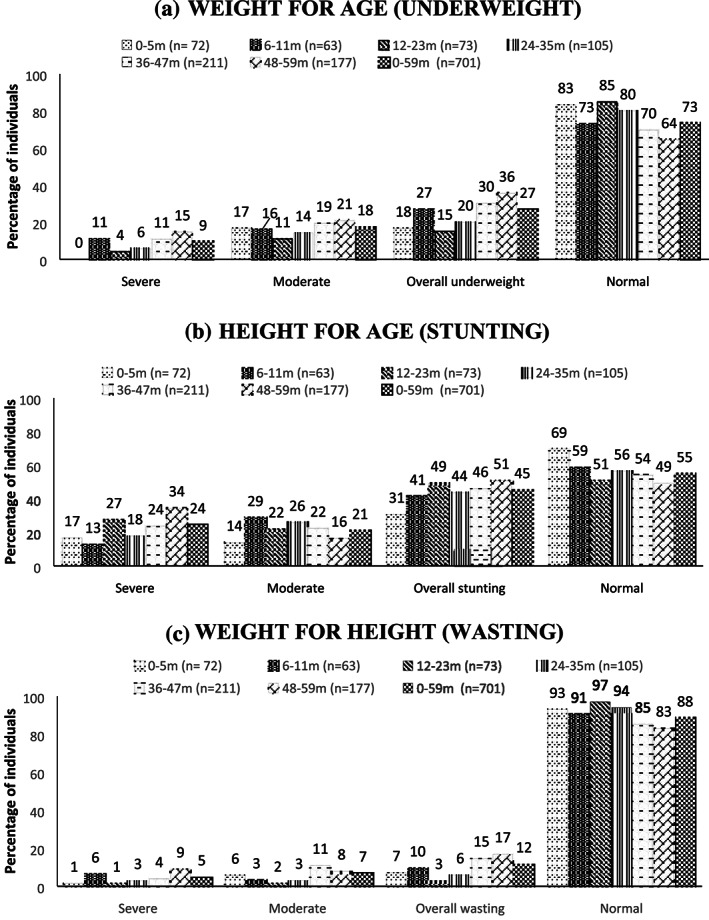
Distribution (%) of children (0–59 months) according to different grades of undernutrition

Figure 1a–c represents the distribution of children below 5 years in terms of underweight, stunting and wasting according to SD classification. Severe underweight was found in all the age groups under 5 years except for the 0–5 months group. The proportion of underweight children below 5 years was 27%, while stunting and wasting was 45% and 12% respectively. Severe stunting was observed in 24% of children below 5 years. Table 1 represents the distribution of individuals in percentage between the age 5 and 17 years and WRA according to the nutritional grades. Among the individuals aged between 5 and 17 years, the age group of 8–12 years had higher proportion of thinness (16%) and stunting (41%) than the other two age groups, that is, 5–7 and 13–17 years (Table 1A). Thinness was observed in 7% and 5% of the individuals in the age groups 5–7 and 13–17 years, respectively. Stunting was observed in the age groups 5–7 years (36%) and 13–17 years (21%). The percentages of overweight individuals were 7% in the age groups 5–7 and 13–17 years, and 8% in 8–12 years of age group. Obesity was found in the age groups 5–7 years (2%) and 13–17 years (1%). Table 1B represents the distribution of WRA in percentages, according to BMI grades. Among these women, 7% were observed to be chronic energy deficient, while the prevalence of overweight and obesity was 24% and 4% respectively

**TABLE 1:** Distribution of individuals according to nutritional grades

1A: Distribution (%) of children 5–17 year‐old individuals according to BAZ and HAZ										
Age group (years)	Nutritional grades									
	BMI Z score						Height for age (stunting)			
	Severe thinness	Moderate thinness	Normal	Over weight	Obesity	Overall thinness	Severe	Moderate	Normal	Overall stunting
5–7 years *n* = 604	9 (1.5)	33 (5.4)	503 (83.3)	45 (7.4)	14 (2.3)	42 (6.9)	65 (10.8)	149 (24.7)	390 (64.5)	214 (35.5)
8–12 years *n* = 852	25 (4.1)	70 (11.6)	463 (76.7)	46 (7.6)	0	95 (15.7)	57 (9.4)	189 (31.3)	358 (59.3)	246 (40.7)
13–17 years *n* = 497	5 (0.8)	24 (4.0)	521 (86.2)	43 (7.2)	11 (1.8)	29 (4.8)	16 (2.6)	108 (17.9)	480 (79.5)	124 (20.5)
1B: Distribution (%) of Women of Reproductive Age (18–49 years), according to BMI grades (WHO classification)										
	BMI grades									
	Chronic energy deficiency (<18.5)	Normal (18.5–24.9)	Overweight (25–29.9)	Obese (≥30)						
WRA (*n* = 1961)	135 (6.9)	1,273 (64.9)	473 (24.1)	80 (4.1)						

*Note.* All values are in *n* (%).

Abbreviations: BAZ, BMI for age Z‐scores; BMI, body mass index; HAZ, height for age Z‐scores; WHO, World Health Organization.

### 3.2 Food consumption

The median daily intake of 13 different food groups by all the categories was calculated and is presented in Table 2. Among the individuals between the age 1 and 17 years, the age group 13–17 years had significantly higher median daily intakes of cereal and millets (520 g), green leafy vegetables (165 g), other vegetables (80.02 g), roots and tubers (105 g), and spices and condiments (13.99 g), compared with the 1–7 and 8–12 years age groups. The daily median intake of pulses and legumes ranged from 26.9 to 52.5 g. While the median daily intake of fish and other sea foods and meat and poultry ranged from 27.51 to 40.95 g and 40 to 50 g, respectively, among the different age groups of individuals aged 1 to 17 years. The WRA had daily median intakes of 580‐g cereal and millets, 63.64‐g pulses and legumes, 166‐g green leafy vegetables and 125‐g milk and milk products. These women's median daily intake of fish and other sea foods and meat and poultry were 33.37 and 252 g, respectively.

**TABLE 2:** Median daily intake of foods and nutrients by the study population

	1–7 y median (IQR)	8–12 years median (IQR)	13–17 years median (IQR)	WRA (NPNL) 15–49 years median (IQR)	*p* value
Food groups					
Cereals & millets (g)	165.94^a^ (125, 227.88)	412.70^b^ (201.92, 500)	520^c^ (504.24, 788.46)	580^c^ (504.24, 788.46)	<0.001
Pulses & legumes (g)	26.92^a^ (16.36, 42.07)	52.50^b^ (32.95, 67.45)	40.38^b^ (26.67, 84.13)	63.64^b^ (26.67, 84.25)	<0.001
Green leafy vegetables (g)	50.02^a^ (29.62, 76.73)	90.80^b^ (76.13, 126.22)	165^c^ (106, 226)	166.49^c^ (116.18, 254.88)	<0.001
Other vegetables (g)	41.18^a^ (26.92, 58.56)	61.35^b^ (46.25, 94.59)	80.02^c^ (76.58, 166.49)	85.82^c^ (56.76, 151.35)	<0.001
Roots & tubers (g)	44.02^a^ (13.72, 70.19)	55.72^b^ (40.99, 97.80)	105^c^ (70.67, 138.72)	110.95 (76.83, 145.98)	<0.001
Nuts & oilseeds (g)	7.21^a^ (6.31, 8.11)	2^b^ (2, 2)	16^ab^ (1.64, 30)	20^c^ (4.15, 20)	0.042
Spices & Condiments (g)	3.37^a^ (1.35, 7.03)	9.19^b^ (4.87, 13.93)	13.99^c^ (8, 17.11)	14.23^c^ (10.18, 18.39)	<0.001
Fruits (g)	10.98^a^ (4.27, 28.38)	31.05^b^ (5.49, 64.29)	42.42^b^ (7.66, 60.65)	25.93^b^ (17.50, 35.17)	0.016
Fish & other sea foods (g)	34.23^a^ (12.99, 38.08)	40.95^a^ (9.13, 53.54)	27.51^a^ (16.97, 57.36)	33.37^a^ (21.88, 51.91)	0.164
Meat & poultry (g)	40^a^ (40, 40)	40^ab^ (40, 92.4)	50^b^ (40, 95)	52.40^b^ (30.23, 75.40)	0.007
Milk & milk products (g)	125^a^ (60, 125)	170^b^ (170, 170)	125^b^ (125, 125)	125^b^ (112.98, 125)	0.004
Fats & oils (g)	4.04^a^ (2.63, 7.50)	7.62^b^ (3.29, 9.17)	7.64^b^ (7.05, 11.38)	7.43^b^ (5.19, 11.12)	<0.001
Sugars (g)	4.76^a^ (3.17, 5)	5.19^b^ (4.15, 6.22)	4.76^b^ (4.76, 7.13)	4.90^b^ (4.76, 7.13)	0.005

Nutrients					
*n*	130	82	98	259	
Protein (g)	29.64^a^ (22.07, 38.67)	58.80^b^ (41.79, 63.01)	73.94^c^ (62.16, 98.16)	76.44^c^ (65.49, 99.85)	<0.001
Total fat (g)	10.08^a^ (6.44, 15.24)	13.01^b^ (7.35, 17.12)	13.07^b^ (6.57, 17.95)	13.51^c^ (9.71, 17.98)	0.005
Carbohydrate (g)	158^a^ (113, 201)	362^b^ (196, 436)	463^c^ (419, 682)	478^c^ (435, 685)	<0.001
Dietary fibre (g)	12.27^a^ (8.91, 16.14)	25.06 ^b^ (18.09, 28.52)	36.71^c^ (29.66, 45.42)	35.95^c^ (30.90, 47.60)	<0.001
Vitamin B1 (mg)	0.37^a^ (0.24, 0.45)	0.72^b^ (0.54, 0.87)	0.97^c^ (0.87, 1.26)	1.01^c^ (0.85, 1.35)	<0.001
Vitamin B2 (mg)	0.48^a^ (0.35, 0.66)	0.90^b^ (0.67, 1.21)	1.36^c^ (1.08, 1.64)	1.39^c^ (1.08, 1.67)	<0.001
Vitamin B3 (mg)	5.06^a^ (3.31, 6.21)	9.75^b^ (7.45, 12.17)	12.94^c^ (11.74, 18.41)	13.96^c^ (12.12, 18.68)	<0.001
Vitamin C (mg)	24.9^a^ (15.58, 42.73)	42.24^b^ (31.49, 53.77)	65.55^c^ (40.37, 101)	68.99^c^ (55.55, 93.34)	<0.001
Vitamin B6 (mg)	0.52^a^ (0.36, 0.65)	0.99^b^ (0.75, 1.26)	1.53^c^ (1.25, 1.75)	1.53^c^ (1.34, 1.97)	<0.001
Vitamin E (mg)	0.63^a^ (0.34, 1.53)	1.26^b^ (0.83, 2.81)	2.73^c^ (1.37, 6.71)	2.63^c^ (1.39, 7.46)	<0.001
Vitamin A (μg)	25.83^a^ (9.33, 83.86)	39.11^b^ (23.20, 151)	60.39^c^ (41.51, 341)	63.61^c^ (30.75, 203)	<0.001
Dietary folate (μg)	127^a^ (88.24, 194)	276^b^ (206, 316)	465^c^ (296, 656)	418^c^ (296, 525)	<0.001
Iron (mg)	6.84^a^ (5.72, 10.94)	14.22^b^ (9.90, 19.67)	22.77^c^ (18.39, 29.24)	22.83^c^ (16.29, 31.06)	<0.001
Zinc (mg)	3.80^a^ (2.86, 4.80)	8.26^b^ (5.25, 8.98)	10.57^c^ (9.49, 14.68)	11.55^c^ (9.69, 15.10)	<0.001
Phosphorus (mg)	509^a^ (346, 638)	845^b^ (519, 967)	1028^c^ (882,1,282)	1119^d^ (910, 1,402)	<0.001
Calcium (mg)	333^a^ (267, 529)	550^b^ (348, 784)	877^c^ (609, 1,024)	905^c^ (728, 1,150)	<0.001
Sodium (mg)	188^a^ (155, 271)	402^b^ (299, 465)	584^c^ (437, 762)	648^c^ (505, 788)	<0.001

*Note.* Interquartile range (IQR) (P_25_, P_75_), where P_25_ is the 25th percentile and P_75_ is the 75th percentile. Median values across the different groups were compared by Kruskal–Wallis test (*p* < 0.05), and the significance differences are indicated by different superscript letters.

Abbreviations: NPNL, nonpregnant nonlactating; WRA, women of reproductive age.

### 3.3 Nutrient intake

Table 2 represents the daily median nutrient intakes of all the individuals in different groups. The median daily intake of protein, carbohydrate and dietary fibre were significantly different between the age groups in children and adolescents. WRA had daily median intakes of 76.44‐g protein, 13.51‐g total fat, 478‐g carbohydrate and 35.95‐g dietary fibre. Dietary daily energy intake of the study groups are presented in Table S2. The daily median intake of energy ranged from 983 (1–7 years) to 2,423 kcal (13–17 years), while WRA had 2,462 kcal. Percentage of energy obtained from the protein, fat and carbohydrate were significantly different among children and adolescents of different age groups. Daily median intake of vitamins and minerals are summarized in the Table 2. The median daily intake of vitamin C, vitamin A and dietary folate ranged from 24.9 to 68.99 mg, 25.83 to 63.61 μg and 127 to 418 μg respectively, among all the groups. The age group 13–17 years had significantly higher intake of all the vitamins analysed, compared with lower age groups 1–7 and 8–12 years. Daily median intake for the minerals iron, zinc, phosphorous, calcium and sodium were calculated (Table 2). The ranges of the median intakes of minerals among the different groups was 6.84–22.83 mg for iron, 3.80–11.55 mg for zinc, 509–1,119 mg for phosphorus, 333–905 mg for calcium and 188–648 mg for sodium. The daily median intake of all the minerals was significantly higher in the age group 13–17 years than in the age groups 1–7 and 8–12 years.

### 3.4 Nutrient adequacy

Adequacy of macronutrients among different age groups of Meiteis was calculated (Table S2) and found that none of the age groups studied satisfied the AMDR. Only 5.4% of the 1–7 years children had intakes within the AMDR. Percentage of energy from carbohydrate (72%–81%) was on the higher side, while energy from fat (4%–10%) was very low. The energy intake from protein was reasonably good (12%–13%). About 52% (1–7 years), 23% (8–12 years), 21% (13–17 years) and 2% (WRA) were consuming <85% of their EER.

**FIGURE 2:**
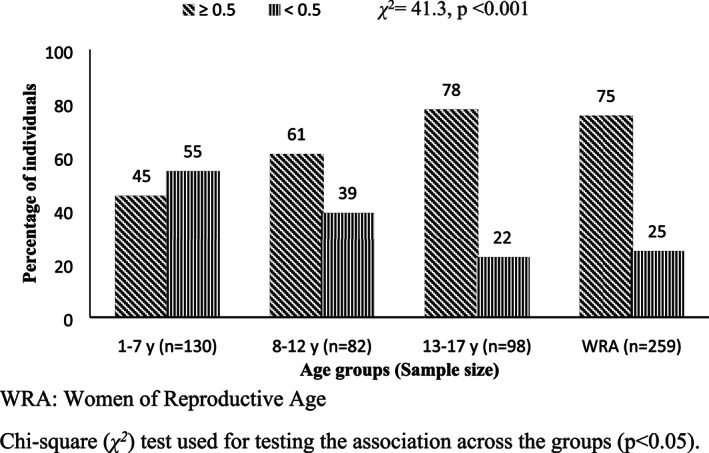
Distribution of individuals based on micronutrient inadequacy (mean probability of adequacy <0.5) *χ* ^2^ = 41.3, *p* < 0.001. WRA, women of reproductive age. *χ* ^*2*^ test used for testing the association across the groups (*p* < 0.05)

The probability of adequacy of micronutrients of the different groups is presented in Table 3. The mean probability adequacy of micronutrients (MPA) was significantly higher in the age group 13–17 years, while it was the lowest in WRA. The PA of vitamins A and E was low in all the groups studied, while PA for calcium was found to be low in the age groups 1–7, 8–12 and 13–17 years. The risk of micronutrient inadequacy (MPA <0.5) in the age group 1–7 years was highest when compared with WRA (*p* < 0.001). Overall, micronutrient adequacy was observed in more than 60% of individuals in the age groups 8–12, 13–17 years and WRA, while it was 45% in 1–7 years (Figure 2), and micronutrient inadequacy was associated with age groups.

**TABLE 3:** Probability of adequacy (PA) and mean probability of adequacy (MPA) among the study population

PA	1–7 years mean ± SD	8–12 years mean ± SD	13–17 years mean ± SD	WRA (NPNL) 18–49 years mean ± SD	*p* value
*n*	130	82	98	259	
Vitamin A	0.06^a^ ± 0.19	0.15^b^ ± 0.34	0.13^b^ ± 0.28	0.13^b^ ± 0.32	0.021
Vitamin E	0.02^a^ ± 0.13	0.001^a^ ± 0.01	0.01^a^ ± 0.10	0.04^a^ ± 0.14	0.08
Vitamin C	0.63^a^ ± 0.46	0.63^a^ ± 0.43	0.60^a^ ± 0.44	0.56^a^ ± 0.44	0.43
Vitamin B1	0.22^a^ ± 0.36	0.56^b^ ± 0.45	0.64^b^ ± 0.41	0.59^b^ ± 0.41	<0.001
Vitamin B2	0.51^a^ ± 0.44	0.66^b^ ± 0.46	0.89^c^ ± 0.23	0.79^d^ ± 0.36	<0.001
Vitamin B3	0.34^a^ ± 0.37	0.62^b^ ± 0.42	0.80^c^ ± 0.25	0.79^c^ ± 0.28	<0.001
Vitamin B6	0.58^a^ ± 0.43	0.70^b^ ± 0.41	0.94^c^ ± 0.18	0.77^b^ ± 0.38	<0.001
Dietary folate	0.41^a^ ± 0.45	0.63^b^ ± 0.43	0.71^b^ ± 0.42	0.66^b^ ± 0.44	<0.001
Calcium	0.13^a^ ± 0.30	0.05^b^ ± 0.18	0.20^a^ ± 0.28	0.62^c^ ± 0.41	0.002
Zinc	0.80^a^ ± 0.34	0.81^a^ ± 0.32	0.95^b^ ± 0.17	0.98^b^ ± 0.11	<0.001
Iron	0.55^a^ ± 0.46	0.75^b^ ± 0.41	0.89^c^ ± 0.27	0.86^c^ ± 0.30	<0.001
Phosphorous	0.68^a^ ± 0.43	0.31^b^ ± 0.42	0.50^c^ ± 0.43	0.96^d^ ± 0.17	<0.001
MPA	0.41^a^ ± 0.24	0.49^b^ ± 0.26	0.61^c^ ± 0.16	0.65^d^ ± 0.72	<0.001

Note. Mean values across different groups were compared by one way ANOVA (*p* < 0.05) with post hoc least significant difference test and the significance differences are indicated by different superscript letters.

Abbreviations: NPNL, nonpregnant nonlactating; SD, standard deviation; WRA, women of reproductive age;.

### 3.5 Dietary diversity score and dietary species richness

Table S4 represents the DDS and DSR across the different age groups. The DDS ranged from 6.23 in 1–7 years to 6.96 in 13–17 years age group; however, there was no significant difference observed among the age groups. In case of DSR, the age group 1–7 years had the lowest (11.85), while other age groups 8–12, 13–17 and WRA had 14.15, 13.39 and 13.55, respectively.

### 3.6 Dietary factors associated with micronutrient adequacy

Logistic regression was carried out to determine the effect of various factors on micronutrient inadequacy and is presented in Table 4. The AOR determines the association of dietary factors with micronutrient inadequacy after the adjustment of the independent variables age and sex, while crude OR (COR) is without the adjustment. The risk of micronutrient inadequacy was significantly higher in the children of 1–7 years age group (COR 3.65; 95% CI [2.35, 5.73]) when compared with WRA. Macronutrient imbalance (AMDR) was observed to have no significant effect on the micronutrient inadequacy. The risk of micronutrient inadequacy was about four times higher when the energy intake was less than 85% of EER. In case of food intake, the lowest tertiles of food groups (except spices and condiments, fish and sea foods and sugars) had higher risk of micronutrient inadequacy. The DDS ≤5 (AOR 1.61; 95% CI [0.86, 3.02]) and DSR ≤13 (AOR 1.88; CI 95% [1.3, 2.72]) had greater risk of micronutrient deficiency. The OR for micronutrient inadequacy decreased for the energy intake less than 85% EER, lowest tertiles of sugar intake, ≤5 DDS and ≤ 13 DSR, when the independent variables age and sex were adjusted (Table 4).

**TABLE 4:** Factors determining micronutrient inadequacy (MPA < 0.5) among study population

Variables		Micronutrient inadequacy (%) (MPA < 0.5)	COR (95% CI)	*p* value	AOR (95% CI) adjusted for age and gender	*p* value
Sex	Male	39.7	1.47 [1.00, 2.17]	0.048		
	Female	30.9	1			
Age (years)	1–7	54.6	3.67 [2.35, 5.73]	<0.001		
	8–12	39.0	1.95 [1.15, 3.3]	0.013		
	13–17	22.4	0.88 [0.51, 1.53]	0.656		
	18–49 WRA	24.7	1			
AMDR	Not satisfied	33.3	1.25 [0.24, 6.49]	0.793	2.52 [0.47, 13.5]	0.281
	Satisfied	28.6	1		1	
Energy intake	<85% EER	61.9	4.61 [2.99, 7.11]	<0.001	3.54 [2.16, 5.81]	<0.001
	≥85% EER	26.1	1		1	
Intake of cereals grains	Tertile‐1	49.6	4.59 [2.77, 7.62]	<0.001	10.2 [5.51, 18.86]	<0.001
	Tertile‐2	22.4	1.35 [0.77, 2.36]	0.298	1.61 [0.87, 2.97]	0.127
	Tertile‐3	17.6	1		1	
Intake Pulses & Legumes	Tertile‐1	42.2	7.75 [3.72, 16.12]	<0.001	10.21 [4.65, 22.4]	<0.001
	Tertile‐2	24.4	3.42 [1.59, 7.37]	0.002	3.55 [1.55, 8.11]	0.003
	Tertile‐3	8.6	1		1	
Intake of green leafy vegetables	Tertile‐1	48.9	4.3 [2.65, 6.97]	<0.001	5.46 [3.26, 9.15]	<0.001
	Tertile‐2	26.6	1.63 [0.99, 2.68]	0.056	1.82 [1.08, 3.06]	0.024
	Tertile‐3	18.2	1		1	
Intake of Roots & Tubers	Tertile‐1	41.6	2.78 [1.73, 4.47]	<0.001	3.37 [2.04, 5.57]	<0.001
	Tertile‐2	35.1	2.12 [1.31, 3.41]	0.002	2.55 [1.55, 4.22]	<0.001
	Tertile‐3	20.4	1		1	
Intake of others vegetables	Tertile‐1	52.2	3.19 [2.05, 4.97]	<0.001	4.17 [2.58, 6.72]	<0.001
	Tertile‐2	19.5	0.71 [0.43, 1.17]	0.176	0.79 [0.47, 1.33]	0.381
	Tertile‐3	25.5	1		1	
Intake of Spices & Condiments	Tertile‐1	32.5	1.68 [1.04, 2.71]	0.033	1.89 [1.15, 3.11]	0.012
	Tertile‐2	41.8	2.5 [1.57, 4]	<0.001	2.67 [1.64, 4.35]	<0.001
	Tertile‐3	22.3	1		1	
Intake of fruits	Tertile‐1	53.7	8.88 [2.3, 34.27]	0.002	16.48 [3.4, 80]	0.001
	Tertile‐2	31.0	3.45 [0.82, 14.53]	0.091	5.25 [1.05, 26.26]	0.043
	Tertile‐3	11.5	1		1	
Intake of Fishes & Other sea Foods	Tertile‐1	30.3	1.04 [0.67, 1.61]	0.874	1.14 [0.72, 1.8]	0.578
	Tertile‐2	37.8	1.45 [0.94, 2.25]	0.092	1.6 [1.01, 2.52]	0.044
	Tertile‐3	29.5	1		1	
Intake of Fats & Edible Oils	Tertile‐1	52.2	3.81 [2.29, 6.33]	<0.001	4.37 [2.58, 7.39]	<0.001
	Tertile‐2	38.2	2.15 [1.26, 3.66]	0.005	2.26 [1.31, 3.92]	0.003
	Tertile‐3	22.3	1		1	
Intake of sugars	Tertile‐1	20.3	0.25 [0.12, 0.53]	<0.001	0.21 [0.11, 0.54]	0.001
	Tertile‐2	21.9	0.28 [0.11, 0.74]	0.010	0.22 [0.08, 0.63]	0.005
	Tertile‐3	50.0	1		1	
Dietary diversity score	≤5	53.1	2.48 [1.37, 4.47]	0.003	1.61 [0.86, 3.02]	0.137
	>5	31.3	1		1	
Dietary species richness	≤13	40.6	2.04 [1.43, 2.92]	<0.001	1.88 [1.3, 2.72]	0.001
	>13	25.1	1		1	

*Note.* As the number of the individuals consuming the food groups namely nuts & oilseeds, milk & milk products and meat & poultry were not adequate for analysis, they were excluded.

Abbreviations: AMDR, acceptable macronutrient distribution range; AOR, adjusted odds ratio; CI, confidence interval; COR, crude odds ratio; EER, estimated energy requirement; MPA, mean probability of adequacy.

## 4 DISCUSSION

This study was carried out to understand the nutritional status (both anthropometric and dietary intake) as well as to identify the dietary determinants of micronutrient inadequacy in the children, adolescents and WRA of Meitei community. The prevalence of stunting in the present study among Meitei children below 5 years was 45%, compared with that of 31% reported in Manipur by NFHS‐4. However, the prevalence of stunting in Meitei children was found to be lower than the in Khasi children (Chyne et al., 2017), and higher than in Chakhesang children (Longvah et al., 2017) from different states of the north‐eastern region of India indicating interstate inequalities within the same region. The prevalence of undernutrition in the Meitei children under 5 years was lower than the national prevalence as reported by NNMB (2012) and NFHS (2015). This could be due to the fact that the status of various determinants, which affects undernutrition among the Indian children under 5 years, that is, use of toilets, low BMI of mothers, acute respiratory infections and diarrhoea in the children (Sinha, Dua, Bijalwan, Rohatgi, & Kumar, 2018), is better in Manipur when compared with the country's status (NFHS, 2015). Among the school‐going children and adolescents of Meitei, the prevalence of thinness, stunting, as well as overweight/obesity was observed in this study. Another study reported by Singh and Devi (2013) also shown the prevalence of underweight and overweight among urban school‐going children and adolescents in the Meitei community. This double burden of malnutrition is currently affecting young children from low‐ and middle‐income countries (Black et al., 2013; Tzioumis & Adair, 2014), and the health of this population has been often neglected (Mokdad et al., 2016). The prevalence of overweight and obesity (28%) was higher than chronic energy deficiency (7%) in the WRA. It is of major concern that overweight and obesity is on the rise in Manipur, which can be seen in the NFHS‐4 report as well. This increasing rise in overweight and obesity needs to be addressed immediately, as it is an important etiological risk factor for many noncommunicable diseases associated with it (Misra & Khurana, 2011). It is established that the increase in the prevalence of overnutrition is driving by the risk factors such as rapid economic growth, urbanization, change in lifestyle and poor nutrition in early phase of life (Abdullah, 2015). This prevalence of double burden of malnutrition in the community will be critical for the policy makers and programme planners as they have to contemplate actions to address both undernutrition and overnutrition simultaneously (Tzioumis & Adair, 2014).

In the present study, it was observed that Meitei households had two major meals in a day, while children indulged in having snacks or small meals in between. Major food comprised rice, vegetables (especially roots and tubers) and fish. The consumption of dairy products, fruits, and fats and oils were low. Gaur and Singh (1994) also observed that the consumption of fruits, eggs and dairy products was extremely low in the Meitei children and also found them with vitamin A deficiency. Most of the adult women and older children (8–17 years) did not consume dairy products, which could be due to existing food habits and low availability of dairy products (Priscilla, Chauhan, & Nagrale, 2016). As milk contains numerous benefits, and in particular it makes a significant contribution to meeting the body's needs for calcium and other essential micronutrients, it is important to increase its consumption among the growing children (Muehlhoff, Bennett, & McMahon, 2013). Moreover, Foote, Murphy, Wilkens, Basiotis, and Carlson (2004) reported dairy products to be a strong predictor of micronutrient adequacy (MPA). It was also found that there was less consumption of nuts and oilseeds, sugars. Consumption of high‐carbohydrate content food groups like cereal and millets and roots and tubers was high in all the age groups. Similar trend of food consumption was also reported in the Khasi children from Meghalaya (Chyne et al., 2017), and Meitei children from Manipur (Gaur & Singh, 1994). This could be the reason for the higher energy intake from carbohydrates than from fat in all the study groups. The traditional fermented and dried fish was consumed on a daily basis and is therefore higher compared with other states of India as reported by NNMB, (2012). This study also found that the low intake (lowest tertiles) of food groups such as cereals and millets, pulses and legumes, green leafy vegetables, roots and tubers, other vegetables, fruits and fats and oils increased the risk of micronutrient inadequacy. Moreover, variation in the food groups can affect the specific nutrient adequacy (Azadbakht, Mirmiran, & Azizi, 2005) as well as overall nutrient adequacy (Torheim et al., 2004). DDS is considered as an indicator of nutrient adequacy (Ogle, Hung, & Tuyet, 2001; Ruel, 2003; Torheim et al., 2004). Recently, it was also reported by Lachat et al. (2018) that DSR to be an appropriate measure for both food biodiversity in the diets as well as micronutrient adequacy. This study observed that DDS ≤5 and DSR ≤13 increased the risk of micronutrient inadequacy. Food biodiversity defined by the diversity of plants, animals and organisms consumed as foods is essential for sustainable food system (Kennedy et al., 2017) as reduced biodiversity has detrimental effects on diet quality and environmental sustainability (Myers et al., 2013).

Most of the individuals did not satisfy the AMDR with high intake of energy derived from carbohydrate, showing imbalanced dietary intake in all the groups. This trend was also observed in WRA of Vietnam, and further reiterated that low education levels, low socioeconomic status and food insecurity are the predictors of imbalanced dietary intake (Nguyen et al., 2013). For individual micronutrients, the probability of adequacies of vitamins A and E were found to be low among all the age groups in the current study. There was also low probability of adequacy for calcium among the individuals of age between 1 and 17 years. Chyne et al. (2017) had reported low intake of vitamin A, calcium and total fat among Khasi children. Similarly, Venkaiah, Damayanti, Nayak, and Vijayaraghavan (2002) also reported a low intake of vitamin A among the rural adolescence girls of India. It has been recently reported that about 68% among south Indian adults (Shalini et al., 2018); and more than 90% among rural adolescent girls and adult women (Radhika et al., 2018) were at the risk of micronutrient inadequacy (MPA <0.5). The inadequate intake of important micronutrients should be a major concern as they are associated with micronutrient deficiencies and its probable ill effects (Ames, 2006). This study identifies the dietary determinants of micronutrient inadequacy in the children, adolescents and WRA of Meitei community, which allow us to understand the factors to be considered for future intervention work in this population. Younger children (1–7 years) of this community were observed to have low intake of most of the major nutrients including energy. Health and nutrition education is necessary for the caretakers to understand the importance of different foods and nutrients and their role in the improvement of children's health. The Meitei community should be encouraged in using nutrient dense foods, which are available to them and should be made aware of their nutritional importance.

## 5 CONCLUSION

This study showed high prevalence of undernutrition among the children of under 5 years, and overnutrition among the WRA was observed in this community. Along with the macronutrient imbalance, the risk of micronutrient inadequacy (MPA <0.5) is also evident in the study population. Therefore, the outcome of this study indicates higher prevalence of malnutrition among the younger children and WRA in this community. In order to improve the maternal and child nutrition, long‐term investments to improve the role of women through education, economic, social and political empowerment is the need of the hour. Enhancing the awareness on the importance of maternal and child nutrition and stimulating a cultural change in favour of a balanced healthy diet with high‐quality locally available foods is necessary for improving future health of this community. Our study used single day 24‐h recall, which does not accurately capture the usual dietary intake long duration; however, it is adequate for estimating group mean intakes of diet (Raina, 2013). The study also covered only 240 households for the dietary recall due to the lack of logistics in the field. Nevertheless, the data obtained highlight the dietary pattern and adequacy, which will allow better understanding for future studies in this community. Our study also failed to capture data on the other confounding variables of nutritional status such as demographics and socio‐economic status.

## CONFLICTS OF INTEREST

The authors declare that they have no conflicts of interest.

## CONTRIBUTIONS

RA, MSR and LT contributed to the study design, NKB was the statistical consultant, and BL was responsible for the field work and drafting the manuscript. All the authors reviewed and approved the manuscript.

## Supporting information

Table S1: List of villages selected for the study

Table S2: Intake of dietary energy per day

Table S3: Food groups considered for the dietary diversity score

Table S4: Dietary diversity score and dietary species richness among the study population
