# Supplementary material for: Dietary adequacy and nutritional status of Meitei community of Manipur, Northeast India
Source: Matern Child Nutr. 2020 Dec 21;16(Suppl 3):e13046. doi: 10.1111/mcn.13046 (PMC7752124; doi:10.1111/mcn.13046)
Supplement: Supplementary file 1 — Table S1: List of villages selected for the study [file MCN-16-e13046-s001.docx]

**Table S1: List of villages selected for the study**

| **Sl. No.** | **Name of the village** | **Sub division** | **District** | **Number of household** | **Population** | **No. of households** | |
| --- | --- | --- | --- | --- | --- | --- | --- |
|  |  |  |  |  |  | **Dietary survey** | **Anthropometry** |
| 1 | WangwooKeirap | Moirang | Bishnupur | 80 | 431 | 3 | 24 |
| 2 | YumnamKhunou | Nambol |  | 251 | 1253 | 7 | 56 |
| 3 | Potsangbam | Bishnupur |  | 804 | 3721 | 23 | 184 |
| 4 | KhaThinunghei | Moirang |  | 1858 | 10107 | 53 | 424 |
| 5 | YaithibiKhunou | Thoubal | Thoubal | 115 | 548 | 3 | 24 |
| 6 | Lourembam | Thoubal |  | 242 | 1050 | 7 | 56 |
| 7 | Lamding | Thoubal |  | 333 | 1587 | 10 | 80 |
| 8 | Nungei | Lilong |  | 392 | 2074 | 11 | 88 |
| 9 | Elangkhangpokpi | Kakching |  | 638 | 2815 | 18 | 144 |
| 10 | Wangjing | Thoubal |  | 672 | 2960 | 19 | 152 |
| 11 | Leishangthem | Lilong |  | 1203 | 6118 | 34 | 272 |
| 12 | Wabagai | Kakching |  | 1810 | 8578 | 52 | 416 |
| **Total** | | | | **8398** |  | **240** | **1920** |
