# Supplementary material for: Dietary adequacy and nutritional status of Meitei community of Manipur, Northeast India
Source: Matern Child Nutr. 2020 Dec 21;16(Suppl 3):e13046. doi: 10.1111/mcn.13046 (PMC7752124; doi:10.1111/mcn.13046)
Supplement: Supplementary file 2 — Table S2: Intake of dietary energy per day [file MCN-16-e13046-s002.docx]

**Table S2: Intake of dietary energy per day**

| **Category** | **n** | **Energy** | | **Energy from carbohydrate** | **Energy from fat** | **Energy from protein** | **AMRD**  **not satisfied** | **< 85% EER** |
| --- | --- | --- | --- | --- | --- | --- | --- | --- |
|  |  | **Kcal** | | **%** | | | | |
| **1-7 years** | 130 | 983^a^  (655, 1133) | | 72.32^a^  (65.05, 76.95) | 9.47^a^  (6.01, 14.72) | 13.34^a^  (11.85, 14.81) | 94.6 | 51.5 |
| **8-12 years** | 82 | 1813^b^  (1227, 2188) | 79.38^b^  (73.94, 81) | | 6.37^b^  (4.48, 8.70) | 12.03^b^  (11.21, 13.13) | 100 | 23.2 |
| **13-17 years** | 98 | 2432^c^  (2106, 3362) | 81.28^c^  (76.76, 82.55) | | 4.02^c^  (2.72, 7.10) | 11.68^c^  (11.10, 12.26) | 100 | 21.4 |
| **WRA 18-49 years** | 259 | 2462^c^  (2195, 3398) | 80.81^c^  (78.88, 82.34) | | 4.66^c^  (3.47, 6.24) | 11.75^c^  (11.11, 12.38) | 100 | 2.3 |
| **P value** |  | <0.001 | <0.001 | | <0.001 | <0.001 |  |  |

EER: Estimated Energy Requirement;

AMRD: Acceptable Macronutrient Distribution Range;

WRA: women of reproductive age; NPNL: Non Pregnant Non Lactating.

All values are median (IQR) except for <85% EER and AMRD

IQR: Inter Quartile Range (P_25_, P_75_), where P_25_ is 25^th^ percentile and P_75_ is 75^th^ percentile.

Median values across the age groups of children were compared by Kruskal-Wallis test (p <0.05), and the significant differences are indicated by superscript letter a, b, and c.
