# Supplementary material for: Dietary adequacy and nutritional status of Meitei community of Manipur, Northeast India
Source: Matern Child Nutr. 2020 Dec 21;16(Suppl 3):e13046. doi: 10.1111/mcn.13046 (PMC7752124; doi:10.1111/mcn.13046)
Supplement: Supplementary file 3 — Table S3: Food groups considered for the dietary diversity score [file MCN-16-e13046-s003.docx]

**Table S3: Food groups considered for the dietary diversity score**

| **Sl. No.** | **Food groups** |
| --- | --- |
| **1** | Cereals and Millets |
| **2** | Pulses and Legumes |
| **3** | Green leafy vegetables |
| **4** | Other vegetables |
| **5** | Roots and tubers |
| **6** | Fruits |
| **7** | Nuts and oilseeds |
| **8** | Milk and milk products |
| **9** | Meat and poultry |
| **10** | Fish |
| **11** | Spices and condiments |
| **12** | Fats and oils |
| **13** | Sugars |
