# Supplementary material for: Dietary adequacy and nutritional status of Meitei community of Manipur, Northeast India
Source: Matern Child Nutr. 2020 Dec 21;16(Suppl 3):e13046. doi: 10.1111/mcn.13046 (PMC7752124; doi:10.1111/mcn.13046)
Supplement: Supplementary file 4 — Table S4: Dietary diversity score and dietary species richness among the study population [file MCN-16-e13046-s004.docx]

**Table S4: Dietary diversity score and dietary species richness among the study population**

| **Nutrients** | **1–7 y** | **8–12 y** | **13–17 y** | **WRA (NPNL)**  **15–49 y** | **p value** |
| --- | --- | --- | --- | --- | --- |
| **n** | **130** | **82** | **98** | **259** |  |
| **DDS** | 6.23^a^ ± 1.20 | 6.89^b^ ± 1.02 | 6.96^b^ ± 0.81 | 6.89^b^ ± 0.95 | 0.032 |
| **DSR** | 11.85^a^ ± 3.10 | 14.14^b^ ± 2.57 | 13.39^c^ ± 2.17 | 13.55^c^ ± 2.42 | <0.001 |

WRA: women of reproductive age; NPNL: Non Pregnant Non Lactating; SD: Standard Deviation; DDS: Dietary Diversity Score; DSR: Dietary Species Richness.

Mean values across different groups were compared by one way ANOVA (p <0.05) with post hoc LSD test and the significance differences are indicated by different superscript letters.
